# 

**Published:** 1882-01-20

**Authors:** 


Abdominal deliveries in the United States for 1880 ... 470
Abortion with retained placenta ... 129
Abortive treatment of buboes ... 268
Acid eructations and heart-burn ... 314
Agaricus in the treatment of night sweats ... 308
Algerian corn cure ... 473
Alcoholic appetite, hereditary ... 262
Amputation of leg, how to perform, by Theodore A. McGraw, M.D ... 54
American public herlth association ... 99
American ash bark ... 115
Amputation of the lower extremities for gangrene from frost-bite, by Thomas F. Houston, M.D., Ga ... 161
Amenorrhœa, treatment of ... 275
American Medical Association ... 390
American inventors, activity of ... 271
American Medical Association, by Dr. Greenley ... 257
Antiseptic solution ... 34
Ants, agricultural of Texas ... 111
Ancient division of time ... 72
Anise seed soothing cordial ... 73
Anæsthetics without witnesses, the dangers of administering ... 146
Antiseptic inhalation ... 150
Antiseptic stimulation in typhoid ... 154
Annual rainfall ... 232
Anti-fat dietary ... 348
Aphrodisiacs ... 75
Aphthous sore mouth in infants ... 434
Aqua ammonia, explosion of ... 150
Aromatic powder of chalk and opium ... 474
Artificial leather ... 152
Arsenic, necrosis from ... 269
Art and industrial exhibition in the capitol at Washington ... 358
Arsenic internally and subcutaneously in the treatment of lymphoma ... 419
Association of American medical colleges ... 37
Asiatic cholera and yellow fever ... 97
Astringent cotton tampons ... 134
Asbestos for stage curtains ... 192
Asthma, fuming inhalations in ... 355
Asthmatic paroxysms ... 433
Atropia, poisoning by ... 146
Ataxi and sewing machines ... 390
Autopsy upon the body of Guiteau ... 264

B

Bacillus lepræ ... 28
Bacteria and diphtheria ... 90
Backward dislocation of the radius and ulna seven months after the injury, successful reduction of, by George E. Brewer, M.D., N. Y ... 253
Baptiste-Jacob, the new Siamese twins ... 272
Bacilli in tubercle ... 308, 377
Bandage after delivery ... 224
Bathing, prolonged, physiological effects of ... 381
Barry, A. L., M. D., something new—nitric acid a remedy for stings and poisons ... 2
Beef tea and coca ... 230
Black sheep in the flock ... 29
Bleeding from the nose, immediate arrest of ... 65
Blood-letting ... 98
Blood enemata ... 180
Blue ointment for ring-worm ... 234
Blackberry extract in diarrhœa ... 355
Book notices ... 39, 78, 157, 199, 317, 359, 399, 438
Bone-conduction of sound ... 60
Boracic acid and calendula in ear disease ... 104
Bovine lymph ... 144
Borax in hoarseness ... 155
Boils ... 236
Bone-felon, treatment of ... 383
Boiling water, curious fact concerning ... 391
Boracic acid ointment ... 435
Bromide ammonia in cholera ... 20
Bromides ... 34, 298
Breast, carcinoma of the ... 108
Bronze coin, a curious ... 392
Bronchocele cured by the hypodermic injection of tincture of iodine ... 414
Bromide of potassium in diabetes ... 427
Butter-making, scientific ... 152
Buboes, abortive treatment of ... 268

C

Calhoun, A. W., M. D., syphilitic ulceration of the eye-lid (conjunctiva) in the infant ... 207
Caustic, nitric acid as a ... 18
Caustic crayon, elastic ... 19
Castor oil ... 19
Carbolic acid, perfumed ... 20
Carbolic spray ... 35
Calomel and chlorate of potash ... 70
Cathartic enema ... 73
Carminative, Dewee's ... 75
Calendula and boracic acid in ear disease ... 104
Carcinoma of the breast ... 108
Carbolic acid in whooping-cough ... 115
Canned meats and vegetables ... 151
Cases in practice, by A. A. Davidson, M.D., Tenn ... 209
Carbuncle, treatment of, by S. Baruch, M.D., N. Y ... 248
Camphorated dover powders ... 275
Calabar bean in obstinate constipation ... 308
Camphorated chloro tannate of iodine ... 313, 353
Cancer, prevalence of ... 316
Cause and effect ... 332
Carbolized nerves as ligatures ... 349
Castor oil as a lactigogue ... 350
Canal on the planet Mars ... 351
Case in practice, by G. A. Cook, M. D., Ga ... 401
Cataract, enucleation and iridectomy ... 422
Cautious ... 432
Cephalic version in the postural position, by John Havenstein, M.D ... 92
Census ... 351
Cerebro-spinal meningitis, treatment of ... 468
Chemical deep-sea soundings ... 472
Chloroforming mice ... 470
Chinese immunity from disease ... 349
Cholera, bromide ammonium in ... 30
Chrysophanic acid concentrated ointment ... 35
Chlorate of potassa mixture ... 35
Chinchona alkaloid, from cuprea bark, a new 110
Chloral and bromide of ammonium in febrile delirium ... 143
Chloroform, death from ... 182, 455
Chloral enemata in vomiting of pregnancy ... 230
Cholera infantum ... 226
Cholera infantum, quinia in the treatment of ... 236
Chorea, arsenical treatment of ... 235
Chordee ... 275
Cholera infantum, by W. F. Hamer, M D ... 292
Chronic dysentery by voluminous enemata of nitrate of silver ... 333
Chloral, large dose of ... 350
Chlorodine, Chandler's ... 383

Chinoidine and capsium in intermittent fever 408
Chinese druggist in New York.......... 417
Cinchonia and quinia.......... 20
Cinchonic mixtures.......... 233
Cinchona alkaloid, a new.......... 390
Climate of Florida.......... 411
Cook, G. A. M. D., Ga., a case in practice.......... 401
Coal, how formed, new theory of.......... 272
Collyrium for metallic foreign bodies on the cornea.......... 30
Comets, tails of.......... 471
Conjunctiva, foreign bodies beneath.......... 184
Cotton seed, to hasten the germination of.......... 31
Cosmical hail.......... 32
Cotton absorbent.......... 33
Coccobacteria in purulent otorrhœa.......... 50
Cough and phthisis.......... 62
Compound fractures and wounds of joints, treatment of.......... 87
Codeia, by J. B. Garrison, M.D., Ark.......... 136
Coated pills.......... 139
Cod-liver oil for young children, disadvantages of.......... 140
Codeia in dysmenorrhœa, sleeplessness and malarial headache.......... 147
Convulsions, nitrate of amylin.......... 142
Conjunctiva to remove foreign bodies from.......... 184
Conjunctiva, application in inflamed.......... 154
Cotton fiber.......... 192
Colicky babies.......... 194
Corn cure, Liebig's.......... 225
Conium, the proper dose of.......... 227
Compression on solids, effect of.......... 231
Constipation, pills for.......... 234
Consumption, its parasitic origin, treatment of, by L. James, M.D., Va.......... 250
Common error that should be corrected.......... 27$
Comedone wash.......... 274
Colostrum as a cause of cholera infantum.......... 312
Constipation.......... 355
Codeine, the value of pure.......... 379
Codeia in diabetes.......... 389
Contagious diseases and pet animals.......... 430
Colon, torpor of.......... 433
Constipation, calabar bean in.......... 308
Constipation, pills for.......... 474
Criteria of insanity.......... 307
Cracked nipples.......... 354
Croupal pneumonia, iodine in.......... 393
Cutaneous eruptions from the use of certain medicines.......... 67

D

Damiana, therapeutic effects of.......... 222
Davidson, A. A., M. D., Tenn.. case in practice.......... 209
Delirium, febrile, chloral in.......... 143
Dentistry, report on progress of.......... 17
Dentaphone.......... 72
Decoction, Zimmerman.......... 75
Delirium tremens.......... 181
Dentistry, mercury used in.......... 263
Diarrhœa pills.......... 413
Dixon, Arch,, M. D., Ky., treatment of malarial coma.......... 165
Disinfactant, new.......... 20
Disinfectant, a new gaseous.......... 32
Diarrhœa of children, cold water enemata in.. 23
Dislocation of the shoulder, to reduce.......... 69
Diphtheria.......... 75
Diphtheria and bacteria.......... 90
Diagnosis wanted.......... 140
Diarrhœa in typhoid..........193, 224
Diphtheria in the past eight years.......... 216
Diarrhœa.......... 265
Diphtheric infection, prevention of.......... 315
Diphtheria.......... 354
Diarhœa, blackberry extract in.......... 355
Diabetes from bromide of potassium.......... 427
Diabetes, codeia in.......... 380
Dreams, controlable.......... 378
Duke, B. F., M. D., Miss., varicose veins of the leg.......... 127
Duties of practitioners in relation to their professional services.......... 218
Durham, Alexander F., M. D., Ga., two cases of lithotomy.......... 1
Dyspepsia from tight bracing.......... 30
Dyspepsia, pills for.......... 235
Dyspepsia and debility.......... 313
Dysphnœa of phthisis and emphysema.......... 314
Dyspeptic vertigo, clinical lecture on.......... 457
Dysentery, treated with aconite.......... 225
Dysmenorrhœa.......... 433
Dynamite.......... 312
Dynamogen.......... 392
Ear diseases, boracic acid and calendula in.......... 104
Earache.......... 183
Earth, tremors of.......... 431
Eczema versus vaccination.......... 148
Eczema, treatment of.......... 233
Eczema, bantigism in.......... 336
Eczema of the genitalia.......... 336
Eczema, excoriated popular.......... 533
Effervescing mixture.......... 115
Elephant, the period of gestation, etc.......... 101
Electric light on the eyesight.......... 112
Electriral experiments.......... 152
Elecric light, naval experiment with.......... 191
Electric light, hygenic value of.......... 191
Elephant in confinement, fecundity of.......... 191
electricity in practice, Thom. F. Houston, on.. 251
Elixir ioda.......... 405
Electric light, novel uses of.......... 432
Emphysema, R. L Hinton, M. D., on.......... 4
Embalmng bodies, a new method of.......... 359
Emergency case of.......... 272
Emmenagogue.......... 394
Enemata of cold water in diarrhœa of children.......... 23
Endometritis, a new method of treating.......... 141
Entropion and trichiasis, Holtz's operation for by A. G. Hobbs, M. D.......... 201
Enucleation and iridectomy.......... 422
Epilepsy, Dr. Hamilton's prescription for.......... 194
Ergotine in pharyingitis.......... 113
Ergot, physical and therapeutical action of.......... 135
Ergot and opium in obstetrics.......... 185
Ergot for lead poisoning.......... 193
Erysipelas, facial, treated by scarification and opium.......... 387
Exematores ulcer of leg.......... 469
Explosive mixtures.......... 24
Expectorants.......... 474
External remedies, formulæ for.......... 74
Extinguishing fire.......... 192
Expert's fees sn court of justice.......... 230
Eye, clinical, remarks on, by A. G. Hobbs.......... 121

F

Faith as an element of cure.......... 464
Fatal chloroform, death from.......... 455
Ferrell, H. V., M. D., practical observations in typhoid fever.......... 47
Filaria sanguinis hominis.......... 04
Flowers in sleeping rooms.......... 111
Fleas, bites of.......... 395
Foreign bodies in the air passages.......... 278
Fracture of the patella.......... 179
Fracture of the odontoid process.......... 458
Freckles, to remove.......... 393

G

Gastric ulcer, iodoform in.......... 267
Garfield doctors' bills.......... 279
Gastrodynia and pyrosis.......... 313
Geum album for gastric irritation and headache.......... 419
Gestation, prolonged.......... 220
Gargle.......... 34
Gizzards and tongues.......... 32
Gonorrhœal pus, the specific germ of.......... 68
Gonorrhœa, by T. H. Logan, M. D., Ga.......... 287
Gonorrhœa.......... 229

Gonorrhœa, injections of sulphurous acid for.. 464
Greenley, T. B., M. D., Ky., variola and vaccina 123
Grease eradicator 194
Grape sugar and glucose 210
Groin cleaning machine 272
Greatness 352
Granular eyelids 394
Green lake 472
Guiteau's body 230
Guiteau, autopsy upon the body of 264
Gum powder as a motive power 471

H

Hail, cosmical 32
Hazeline, as a local application in irritable and inflamed piles 110
Hair tonic 152
Hay-faver, treatment of 221
Hamer, W. F., M. D., on cholera infantum 292
Hayes Bros., M. D., Ark., clinical report of a compound comminuted complicated fracture, etc 281
Headache, frontal, iodide of potassium in 106
Herpes zoster 114
Hemicrania 153
Hendree, J., M. D., midwifery extraordinary 444
Heart, hot water for 236
Heartburn and acid eructations 314
Hermophradite 389
Henry, F. P., M. D., remarks on intestinal porasites 113
Hinton, R. L., M. D., Ark., recurrent malarial attack, 3; emphysema, 4; rhus toxidendrom, 41; Trade-mark and copyrights, 126; case in practice 323
Hobbs, A. G., M. D., remarks on Holtz's operation for entroion and trichiæsis, with cases, 201; otalgia from reflex dental irritation 321
Houston, Thomas F., M. D., Ga., viburnum prunifolium, 41; amputation of the lower extremities for gangrene from frostbite, 161; electricity in its relations to general practice 242
Hoarseness, borax in 155
Hope's camphor mixture 234
Humerus, dislocation of, new method of reducing 469
Hydrocele, method of treating 440
Hypodermic syringe 29
Hypodermic injection, improvement in 30
Hypodermic injection of water in the treatment of pain 67
Hypodermic injection of quinine 68
Hydrophobia cured 389
Hydrate of chloral and tincture iodine 389
Hydrophobia, recovery of nine cases of 427
Hydrocinate of iron in neuralgia 295
Hypodermic syringe, to keep 463

I

Improvement in hypodermic injection 30
Infants, feeding of 337
Ingrowing toe-nail, select formula for 26
Injection Brou 34
Information wanted, by A. G. Smyth 128
Incontinence of urine 177, 314
Infantile diarrhœa 186
Intestinal parasites, remarks on, by F. P. Henry, M. D 213
Insanity, criteria of 307
Inflammation of the testis, inflammation of pulsatilla in 330
Intermittent fever and quinine hypodermically 349
Intussusception, treatment of 428
Inundations on planet Mars 432
Inhalations in asthma 355
Ins and outs 475
Intestinal investigation, puncture and aspiration in 467
Iodoform, odorless 21
Iodoform in gynecological practice 153
Iodoform in gastric ulcer 267
Iodoform as a dressing to fresh wounds, by Lindsay Johnson, M. D., Ga 285
Iodoform in ulcers 314
Iodoform in lung diseases 315
Iodide of potassium in frontal headache 106
Iodine in typhoid fever 193
Iodine, camphorated chlro-tannate of 313
Iodine in croupal pneumonia 394
Iron albuminate 20
Itching, remedy for 34

J

Jamaica dogwood 229, 355
Jaborandi 210
Johnson, Lindsay, M. D., Ga., iodoform as a dressing to fresh wounds 285
Journalism in the south 239

K

Kelly's tonic 114
Koch at the German congrees 300
Kouso 19

L

Laryngeal phthisis 108
Lappa major 415
Leucorrhœa 153
Lead poisoning, ergot for 193
Lead colic, relief of pain in 227
Letter, Dr. Reiter's 415
Lithotomy, two cases of, by A. F. Durham, M. D 1
Lithotomy, supra-pubic operation for 27
Life in other planets 31
Listerine 337
Locusts as food 151
Logan, T. H., M. D., Ga., gonorrhœa 287
Lung disease, iodoform in 315
Lutidine as an antidote for strychnia 307

M

Magnetic observations 471
Malarial fever, recurrent, by R. L. Hinton, M. D 4
Malaria, what is it 29
Malarial chill, treatment of 155
Malaria in skin diseases, a correction 466
Malarial coma, treatment of, by Arch Dixon, M. D., Ky 165
Malaria treated with the tincture of iodine 306
Magnifera indica or mango 340
Materialists 392
McConnell, R. F., M. D., Ala., veratrum viride in puerperal convulsions 361
Meats and vegetables, canned 151
Menstrual flow at age of seventy 153
Medical Association of Georgia 196
Mercury used in dentistry 268
Membranous dysmenorrhœa, prescription for 270, 395
Metallic writing pencils 272
Metal, new 471
Mercurial salivation 274
Medical colleges and quackery 276
Medical literature in the south 477
Medical progress 436
Mesmerizer, traveling, shall he be abolished 382
Microscopists at dinner 472
Milk to extinguish petroleum 113
Mixture, anti-rheumatic 115
Mixture, effervescing 115
Micro organism and disease 229
Milk, illness caused by filth in 256
Microscopy and pathology 261
Mild secretion, influence of certain remedies upon 310
Mistletoe in affections of the heart 350
Mica masks 391
Mistura opii composita 394
Midwifery extraordinary, by J. Hendree, M. D., Ala 444
Morphia in puerperal eclampsia 69

Movement as a means of cure .......... 83
Motive power, transmission of .......... 311
Mosquito oil .......... 205
Mortality of whites and blacks in the United States army .......... 476
Muriate of ammonia wash .......... 35
Mosquito as a carrier of disease .......... 71
Mustard in the treatment of small-pox .......... 468

N

Nasal douche, evil effects of .......... 374
Naguet's hair-dye, modified .......... 474
Necrosis from arsenic .......... 269
Neuralgia .......... 269 395
Neuralgia and its treatment, practical notes on .......... 424
Nervine and anti-spasmodic .......... 434
Nitric acid, a remedy for stings and poisons, by A. L. Barry, M. D .......... 2
Nitric acid as a caustic, method of applying... 18
Night sweats .......... 63
Nitrate of amyl in convulsions .......... 142
Niagara, rate at which it recedes .......... 231
Night sweats, agaricus in the treatment of .......... 308
Nitrous oxide .......... 420

O

Obstetrics, ergot and opium in .......... 185
Obstinate constipation, calabar bean in .......... 308
Obstetrical experience, an interesting .......... 335
Oil of turpentine in mixtures .......... 235
Ointment, Hardy's .......... 235
Oil of wintergreen, as an effective salicylate in rheumatism .......... 110
Opium habit, solid extract of coca in, by John Q. Winfield, M.D., Va .......... 9
Otalgia from reflex dental irritation, by A. G. Hobbs M.D .......... 321
Oxyuris vermicularis .......... 265
Ozæna, treatment of .......... 145
Ozone as a sleep-producing agent .......... 182

P

Parafine ointments and oleates .......... 19
Parasiticide .......... 35
Pamphlets, etc .......... 38
Pain, hypodermic injection of water in the treatment of .......... 67
Parturition among the Abyssinians .......... 469
Pasteur, M .......... 152–232
Patella, fractures of the .......... 179
Paracentesis of the bladder through the perineum and prostate .......... 189
Parasitic nature of tubercular consumption... 219
Pathology and microscopy .......... 261
Parsley as an antigalactic .......... 150
Pet animals and contagious diseases .......... 430
Pharyngitis, ergotine in .......... 113
Physician, what constitutes a good .......... 28
Phthisis and cough .......... 62
Physicians who do not read .......... 147
Phthisical cough, treatment of .......... 155
Phimosis and adherent prepuce, consequences of .......... 189
Phthisis, laryngeal, treatment of .......... 234
Photo-printing in colors .......... 312
Phagedenic ulcers, treatment of .......... 313
Phenic acid in yellow fever .......... 428
Physicians, legal responsibility of .......... 176
Piles, hazeline as a local application in .......... 110
Piles, aloes for .......... 113
Pitting in smallpox, to prevent .......... 193
Pimples on the face, sulphur for .......... 183
Plaster of Paris in club-foot .......... 5
Planets, life in .......... 31
Placenta, delivery of .......... 66, 406, 421
Plank sidewalks, their effects upon health .......... 238
Placenta prævia .......... 370
Poultice, new .......... 19
Potash, calomel and chloral .......... 70
Poison of rattlesnake .......... 70
Potassium permanganate as an antidote to the venom of serpents .......... 95
Poisoning by atropia .......... 146
Podophyllin, overdose of .......... 222
Poisoning by Winslow's Soothing Syrup .......... 268
Post-partum hemorrhage, tincture of perchloride of iron in .......... 368
Poisoning from red stockings .......... 429
Population, centre of .......... 392
Pruritus vulvæ .......... 33
Prophylaxis of smallpox .......... 150
Prize of $1,000 .......... 185
Pregnancy, vomiting in .......... 223–402
Pruritus ani .......... 228
Preserving meat .......... 271
Practice, a case in, by R. L. Hinton, M.D .......... 323
Pregnancy at the age of sixty-two .......... 384
Premature children, viability of .......... 429
Principles of inhalation .......... 385
Puerperal eclampsia, use of morphia in .......... 69
Pulmonary phthisis, curability and treatment of .......... 102
Pulmonary gymnastics .......... 112
Puerperal eclampsia .......... 171
Purpura .......... 314
Pulsatilla in inflammation of the testis .......... 330
Pulvis doveri .......... 460
Purdy, A. M. E., M.D —Viburnum opulus in dysmennorrhœa and uterine pain .......... 445
Pyrogallic acid .......... 21
Pyrosis and gastrodinia .......... 313

Q.

Quicksilver, a dose of .......... 345
Quinia and cinchonia .......... 20
Quinia in treatment of cholera infantum .......... 236
Quinine .......... 138
Quinidia, combination for intermittents .......... 236
Quinine, bad effects of .......... 190
Quinine, hypodermic injection of .......... 68
Quinine, hypodermically for intermittent fever .......... 349
Quinine, effects on intra-cranial circulation .......... 347

R.

Rabies with hoang-nan, treatment of .......... 227
Rabies, Pasteur on .......... 344
Rattlesnake poison .......... 70
Rapid Pulse, how to count .......... 303
Replacing and healing of pieces separated from the human body .......... 364
Retention of placenta .......... 366
Rhus toxicodendron, by R. L. Hinton, M.D., Ark .......... 43
Rheumatism, oil of wintergreen in .......... 110
Rheumatic mixture .......... 115
Rheumatism, acute and chronic .......... 153
Rheumatism, acute .......... 109
Ring-worm .......... 275
Rotheln, rubeola or German measles .......... 149
Rust, to preserve iron from .......... 31

S

Salicylic acid, ointment of .......... 35
Salts, Preston's .......... 73
Sabal—serrulata—saw-palmetto .......... 141
Salicylic acid, vehicle for .......... 221
Salicylate of potassa in acute rheumatism and dyspepsia .......... 346
Salicylic cream .......... 394
Sanitary convention .......... 398
Salicylate of sodium incompatible with spirits of nitrous ether .......... 230
Scarlatinous virus, permanence of .......... 188
Scrotal pruritus .......... 194
Sciatica, injection for .......... 313
Self-abortion .......... 11
Sea-sickness, treatment of .......... 104
Sexual abnormalities .......... 137
Sensible .......... 310-463
Secrets of our patients .......... 347
Size of drops, comparative .......... 388
Singultus, a specific for .......... 435
Skin grafting .......... 461
Sleeping rooms, temperature of .......... 27

Sleeplessness, remedies for......... 296
Sleep, new method of inducing......... 301
Sleep-producing agent......... 425
Small-pox pustulation in the fœtus in utero... 59
Small-pox.........76-100
Small-pox and vaccination......... 103
Small-pox, prophylaxis of......... 150
Small-pox, to prevent pitting after......... 193
Small-pox, Wight on the combating of......... 228
Small-pox, vaccination after......... 270
Small-pox and Scarlatina in same subject...... 430
Small-pox in birds......... 430
Small-pox, mustard in......... 468
Smythe, A. G., M.D., Miss.—Viburnum prunifolium unreliable in abortion—a rejoinder, 81—Information wanted......... 128
Snake-bite, treatment of......... 345
Soap liniment......... 473
Sound, bone conduction of......... 60
Southern medical college, commencement..... 116
Spleen, extirpation of......... 420
Spermatorrhœa, treatment of......... 29
Sponge grafting......... 52
Story on old Doctors......... 7
Strychnia, lutidine as an antidote for......... 307
Stings and poison, nitric acid a remedy for..... 2
Stammering and its cure......... 15
Stimulating lotion......... 35
Styptic colloid......... 113
Strychnine, antidote for......... 226
Stomach, washing of......... 254
Stomach, position of......... 304
Suberin......... 20
Supra-pubic operation for lithotomy......... 27
Sulphur......... 32
Sulphide of calcium in strumous ophthalmia, 105
Sulphur for pimples on the face......... 183
Sudden death during forced depression of tongue......... 187
Suit against a doctor......... 187
Sulphurous acid in treatment of gonorrhœa... 464
Surgery in the new college......... 476
Surgery, Dr. Bird on......... 266
Surgery, oral and dental......... 270
Sunstroke......... 304
Surgery and the doctrine of evolution......... 341
Surgeons, German, Congress of......... 309
Sugar, whitening of......... 192
Syringe, hypodermic......... 29
Syphilis, vaccinal......... 46
Syphilis.........114, 430
Syphilis without mercury......... 178
Syphilis, ulceration of the eye-lid in the infant, by A. W. Calhoun, M.D., Atlanta... 207
Syphilis, can a man have it twice?.........228, 380
Syphilitic infection of the finger by medical men......... 267
Sweating, excessive......... 388

T

Tape-worm. new remedy for......... 394
Teeth, extraction of during pregnancy......... 470
Telephone sound of voices......... 71
Tetanus cured......... 188
Teeth, cause of the decay of......... 230
Temperature, rise in......... 232
Tea and coffee, the therapeutics of......... 245
Thoracic duct in the case of President Garfield, 21
Theory of disease, Huxley's......... 154
Therapeutics, hot water in......... 297
Thapsia gargarica, effect of on the skin......... 309
Tight lacing, dyspepsia caused by......... 30
Tincture of iodine, Churchill's......... 35
Tincture of iodine, malaria treated by......... 306
Tincture of perchloride of iron in post-partum hemorrhage......... 368
Tonsillitis treated by ice and quinine......... 268
Toe-nail, select formulæ for......... 26
Tongues and gizzards......... 32
Tonic bitters, mild......... 114
Tonic, Hamilton's......... 115
Tonsillotomy by ignipuncture......... 386
Tonsillitis......... 393
Transplantation of muscle from the dog......... 470
Transactions Medical Association of Georgia.. 77
Trademarks and copyrights, by R. L. Hinton, M.D......... 126
Trichinæ, do Southern hogs have......... 183
Trichinæ, degree of heat to destroy......... 232
Tubercular consumption, the parasitic nature of......... 219
Typho-malarial fever......... 477
Typhoid fever, practical observations in......... 47
Typhoid fever, cold applications in......... 61
Typhoid, antiseptic stimulation in......... 154
Typhoid fever and diarrhea, iodine in......... 193
Typhoid fever, antiseptic treatment of......... 465

U

Ulcers, chronic......... 274
Ulcers. iodoform in......... 314
Urethritis, is it a specific disease?......... 25
Urine, incontinence of.........177, 314
Urinary analysis, recent advances in......... 388
Urine, disinfection of......... 390
Urticaria, epidemic......... 396
Uterine hemorrhage, alum plug in......... 149
Uterine fibroids.........234, 273
Uterine hemorrhage, a new method of treating......... 373
Uterine hemorrhage, two weeks after delivery, a case of, by T. B. Greenley, M.D., Ky. 441

V.

Vaccination, animal......... 10
Vaseline, carbolized......... 35
Vaccinal syphilis......... 46
Vaccination and vaccinization......... 57
Vaccination, does it protect?......... 64
Vaccination and smallpox.........103, 270
Vaccination, questions on......... 107
Vaccination in Scotland......... 109
Variola and vaccinia......... 123
Varicose veins of the leg, by B. F. Duke, M.D., Miss......... 127
Vaccination versus eczema......... 148
Vaccine farms, report on......... 168
Vaginitis, new treatment for......... 236
Vaginitis, treatment for......... 274
Variola, susceptibility to......... 59
Vegetables and canned meats......... 151
Vesico-vaginal fistula......... 302
Veratrum viride in puerperal convulsions, by R. F. McConnell, M.D......... 361
Venereal disease, the rational and routine treatment of......... 378
Vesical calculi in children, detection of......... 389
Vesico-vaginal fistula cured by position......... 462
Vegetable liver pills, Dr. Yuna's......... 435
Vertigo, a clinical lecture on......... 457
Viburnum-prunifolium, by Thomas F. Houston, M.D......... 41
Viburnum prunifolium unreliable in abortion—a rejoinder—by A. G. Smythe, M.D. 81
Viscum album, or mistletoe, in affections of the heart......... 350
Viability of premature children......... 429
Viburnum-opulus in dysmenorrhea and uterine pain, by A. M. Purdy, M.D......... 445
Vomiting in pregnancy, Chloral enemata in.... 230
Vomiting in pregnancy.........228, 402

W

Warts, removal of......... 109
Whooping-cough, carbolic acid in......... 115
Wine-marks, treatment of......... 106
Winslow's soothing syrup, poisoning by......... 268
Wine, digestive......... 306
Worms crawling under the skin......... 70
Worms, smartness of......... 352

Y

Yellow fever and Asiatic cholera......... 97
Yellow-fever, phenic acid in......... 428
Yerba santa, physiological action of......... 133

### *RECEIPTED.*

1881.—Drs. G W Bowling, W E Bryant, J W Talley, B F Darnell, A H Smith, R J McMullan, W B Maxwell, J H Goss, Colin Bethune, B M Woolley, H H Malone.

1882—Drs. R H Edwards, N G West, J E Wright, J W Harris, W B King, N B Warren, E Wheeler, E A Anderson, J F Blanks, A R Jones. W T Beall, R S Powell, J H Wysong, S V Pankes, W J Lee, R A Shimpoch, A Atkinson, W A Brown, A B Loving, Dr. Chub, P C Tircuit, J W Vissage, C B Thomas, C H Smith, S M Hogan, J E Fripp, R F Mathews, E H Parham, Lockwood Alison, R L Hinton.

